# Florfenicol sustained-release granules: an *in vitro-in vivo* correlation study in pigs

**DOI:** 10.1186/s12917-023-03631-2

**Published:** 2023-06-30

**Authors:** Wei-cong Yang, Zi-yao Liu, Yun-xiao Zhang, Yang Yu, Yue Shen, Ying Xu, Xian-hui Huang

**Affiliations:** 1 Veterinary Pharmacology and Toxicology, College of Veterinary Medicine, South China Agricultural University, 483 Wushan Road, Tianhe District, Guangzhou, 510642 China; 2 National Risk Assessment Laboratory for Antimicrobial Resistance of Animal Origin Bacteria, Guangzhou, 510642 China

**Keywords:** Pharmacokinetic, Oral delivery, In vitro-in vivo correlation, Pig, Florfenicol

## Abstract

The objective of this study was to synthesize and characterize pharmaceutical characteristics of florfenicol sustained-release granules (FSRGs) in vitro and in vivo. FSRGs were synthesized using monostearate, polyethylene glycol 4000 and starch. In vitro dissolution profiles were studied using the rotating basket method in pH 1.2 HCl solution and pH 4.3 acetate buffer. Twenty-four male healthy Landrace×Yorkshire pigs were equally divided into three groups and administered a 20 mg/kg *i.v* bolus of florfenicol solution and dosed orally with FSRGs in the fasting and fed states. The Higuchi model was the best fit for the drug release profile in pH 1.2 and pH 4.3 media, and the mechanism of drug dissolution was governed by both diffusion and dissolution. We established a level A in vitro - in vivo correlation for FSRGs and the in vivo profile of the FSRGs can be estimated by the in vitro drug release.

## Introduction

**Fig. 1:**
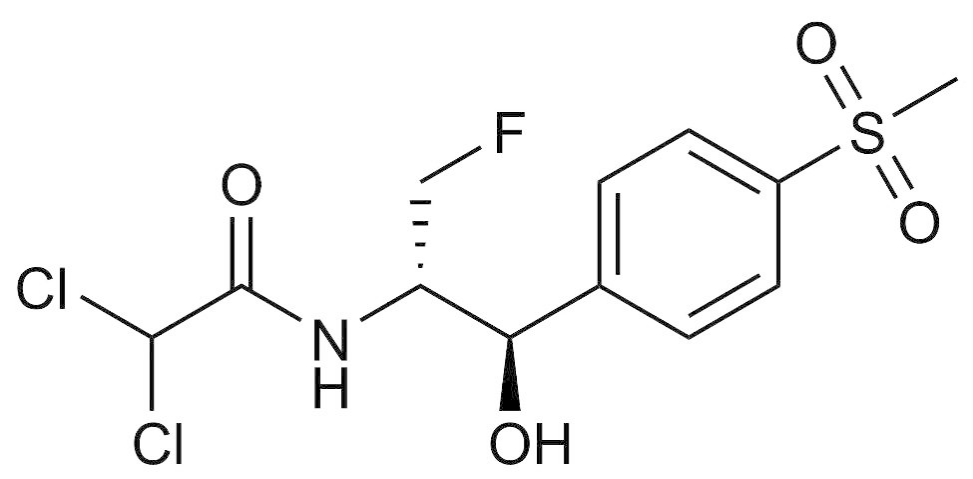
Chemical structure of florfenicol (C12H14Cl2FNO4S; CAS 73231-34-2)

Florfenicol (FFC) is a broad-spectrum bacteriostatic antibiotic that inhibits protein synthesis by binding to the large ribosomal subunit of the bacteria. These properties make the drug effective in treating bacterial infections of farm animals and aquaculture [1] (Fig. 1). FFC exhibits high activity against pathogens responsible for massive livestock losses, i.e. primary respiratory pathogens such as *Mycoplasma spp.*, *Pasteurella multocida* and *Actinobacillus pleuropneumoniae*, as well as gastrointestinal pathogens such as *Escherichia coli* and *Salmonella* [1–4]. High concentration of FFC in the pulmonary epithelial lining fluid and lung tissue makes it a widely used antibiotic in the treatment of respiratory diseases [5–7]. High system bioavailability and tissue concentration make it effective in the treatment of *Escherichia coli* infections in broilers [8–10].

Sustained-release preparation of a drug has considerable advantages of slow and sustained release of the drug, as well as the reduced fluctuation of its concentration in the blood. The Sustained-release granules can be described as a formulation in which active pharmaceutical ingredients are dispersed or coated by medical polymer materials, and that has pharmaceutical properties of controlled release and enteric solubility [11–13]. Over the past decade, there have been a large number of research programs on FFC sustained-release formulation that used nanotechnology and high-molecular compounds as ingredients [14–18]. Most of them, however, must be administered by an injection, which is less suitable for large-scale farming. Quick and easy administration, good acceptance by livestock and low cost, render oral administration the main and simplest way of prevention and treatment of a large group of animals.

Spray-drying has been used for decades in medicine and food industry. The process is mainly used for formulating small hydrophobic molecule drugs, large biomolecules and biopharmaceuticals [19, 20]. More importantly, spray-drying is advantageous over other preparation technologies due to its relatively low cost, energy consumption and scale-up potential.

Most current dosage forms of commercial FFC for oral administration in China are immediate-release forms such as FFC powder and FFC premix. In this study, we chose monostearate (controlled release), polyethylene glycol (PEG) 4000 (erosion-controlled release) and starch (filler) as excipients [21, 22]. The objective of this study was to develop a florfenicol sustained-release granules (FSRGs) formulation and investigate the in vitro release of FSRGs and its pharmacokinetics in pigs.

In this study, we prepared FSRGs using high-speed centrifugal spray-drying and examined its in vitro release and pharmacokinetics in pigs.

## Results

### Surface characterization and stability of FSRGs

**Fig. 2:**
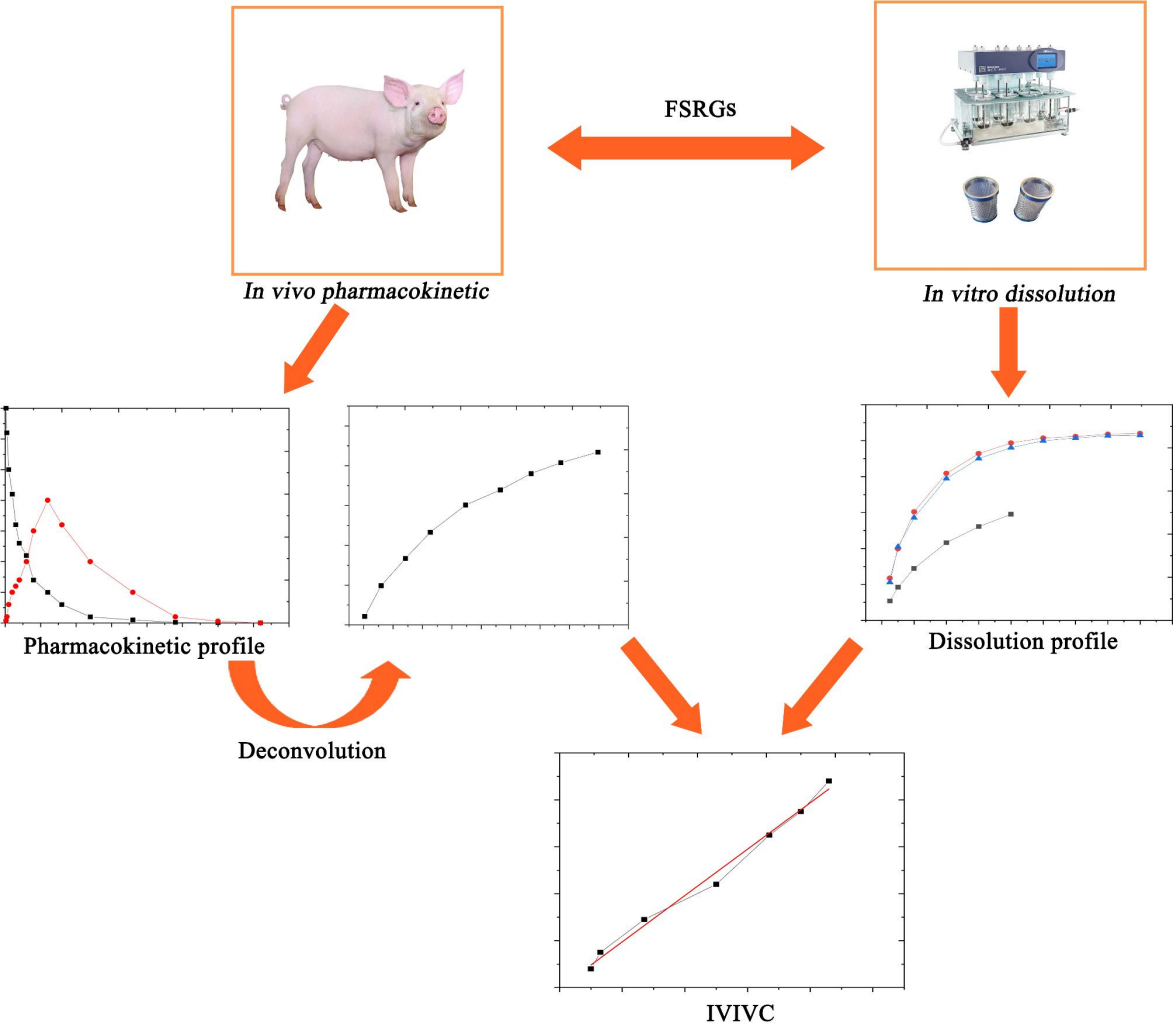
Schematic flow in to establish an *in vitro* and *in vivo* correlation for florfenicol sustained-release granules

**Fig. 3:**
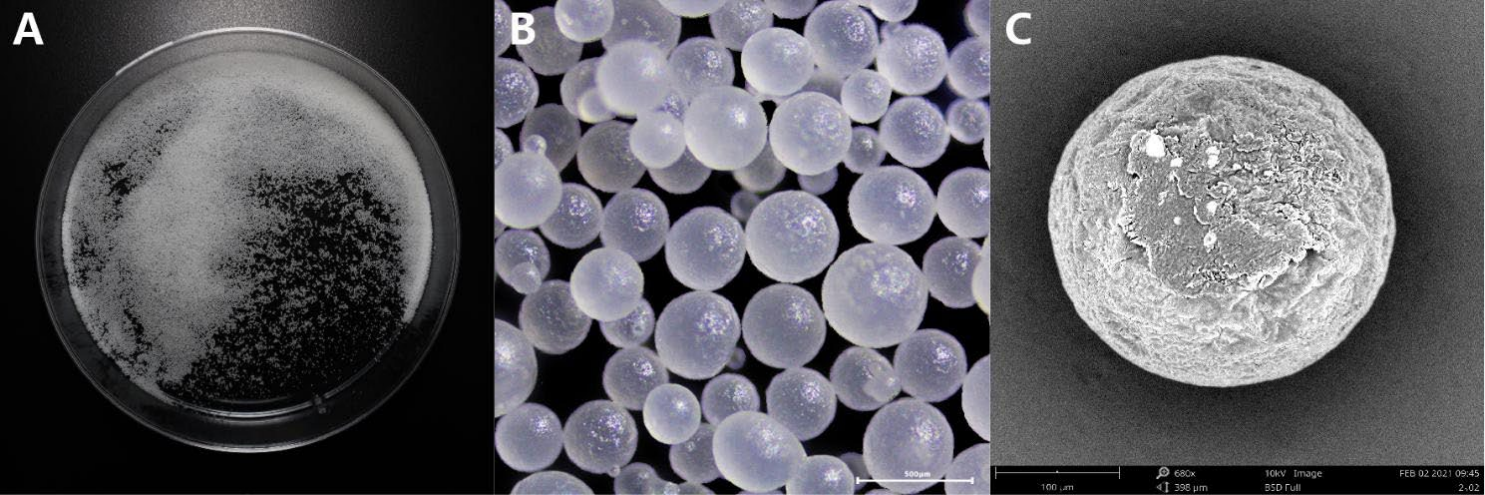
Structural characterization of Florfenicol sustained-release granules. **A**, Appearance status of FSRGs; **B**, Light micrographs of FSRGs, 40×magnification; **C**, Scanning electron micrograph of FSRGs, 680×magnification

The FSRGs we synthesized were small, pale white, and loose, non-clustering granules that could be separated by shaking the vessel slightly (Fig. 2A). The particles were spherical of relatively smooth surfaces and were only minimally transparent (Fig. 2B C). The florfenicol content of FSRGs was 10.3%. Most FSRGs particle sizes (more than 99.5%) were within 600 μm in diameter and the particle size distribution is presented Fig. 3. FSRGs were stable in the influencing factors test and accelerated test, and there were not any significant changes of color, particle shape and content (less than 1% was lost), as the influencing factors test and accelerated test revealed. There was no observed hygroscopicity (data not shown).

### In vitro release study

**Fig. 4:**
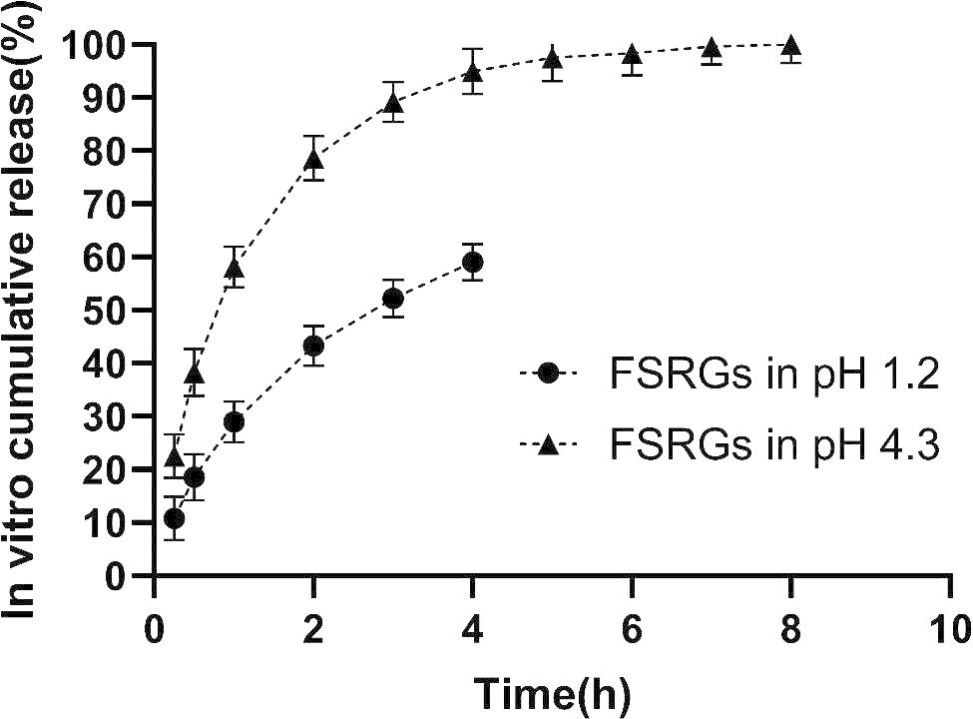
Particle size distribution of Florfenicol sustained-release granules

The in vitro cumulative release rates of FFC from the FSRGs after 4 h was 59 ± 1.5% in pH 1.2 solution as compared to 71.8 ± 1.1% at the same time and over 90% after 8 h in pH 4.3 solution (Fig. 4). The best model that fit the data was the Higuchi model for both pH 1.2 and pH 4.3 data sets (Table 1).

### In vivo study and pharmacokinetic parameters

**Fig. 5:**
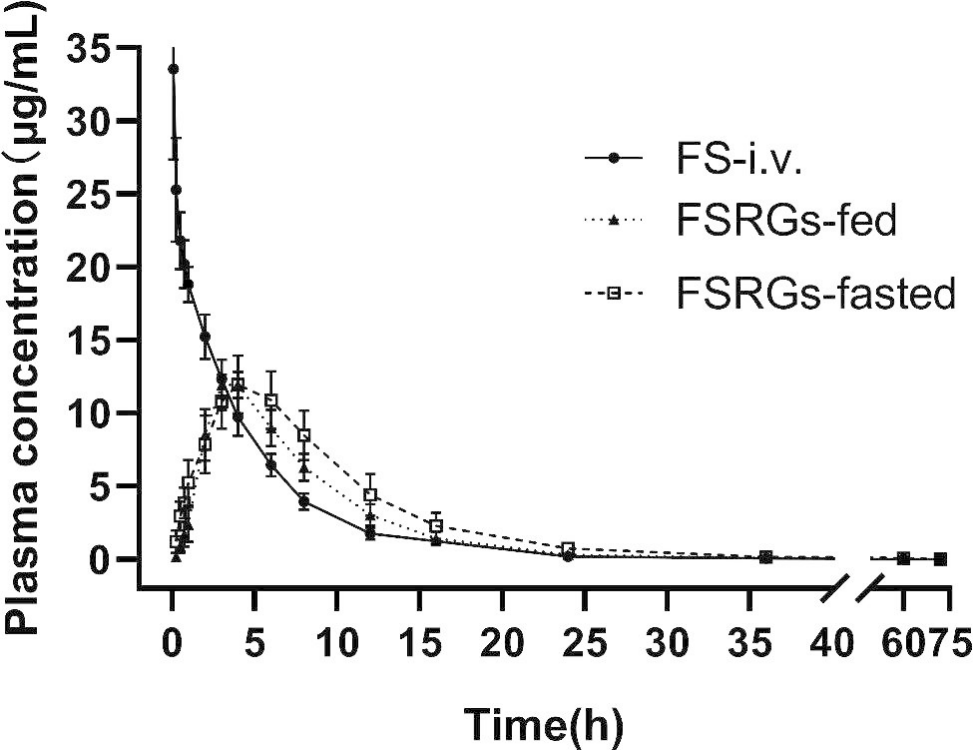
*In vitro* dissolution profile of florfenicol released from FSRGs in pH 1.2 HCl solution and pH 4.3 acetate buffer. Data were expressed mean ± SD (n = 3), error bars represent standard deviations

We measured the pharmacokinetics of FSRGs in vivo in pigs that were fasting and fed, and compared the results with those of the groups receiving the formulated FFC solution as an i.v. bolus in non-fasting conditions. All the pigs were clinically normal throughout the study. The mean plasma concentration versus time curve of each group after administration of FFC is shown in Fig. 5. Table 1 lists the calculated pharmacokinetic parameters as mean ± standard deviation (n = 8).

**Table 1:** Fitting results of florfenicol release from FSRG

Dissolution media	Release kinetics model	Fitting equation	Coefficient of determination (r^2^)
pH1.2 HCl	Zero-order	$Q=17.156t$	0.7532
	First-order	$\ln (100 - Q)= - 0.262t$	0.9240
	Higuchi	$Q{\text{=}}29.491{t^{1/2}}$	0.9862
pH 4.3 acetate buffer	Zero-order	$Q=13.952t$	0.5124
	First-order	$\ln (100 - Q)= - 0.344t$	0.9508
	Higuchi	$Q{\text{=}}34.095{t^{1/2}}$	0.9775

Zero order($Q={k_0}t$), first order($\ln (100 - Q)={k_1}t$), Higuchi($Q{\text{=}}{k_H}{t^{1/2}}$)

### In vitro-in vivo correlations

**Fig. 6:**
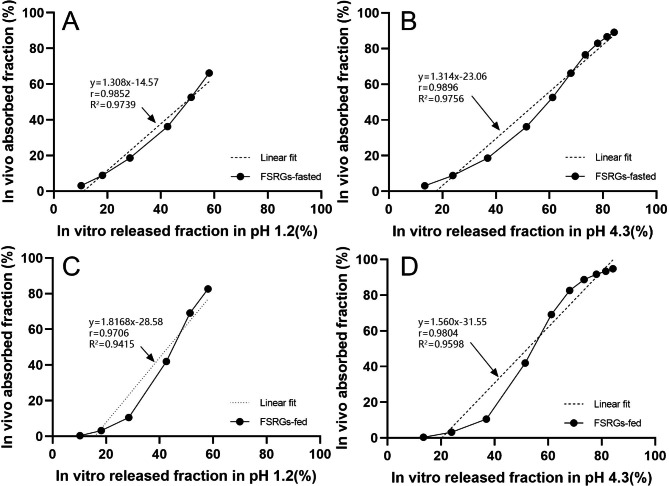
Mean plasma concentration versus time profiles of FSRGs administrated at fasted (hollow square) and fed (solid triangle) condition, and florfenicol solution (solid circle) after i.v. in pigs. Data were expressed mean ± SD (n = 8), error bars represent standard deviations

The correlation between in vivo and in vitro data was the strongest with FSRGs in the fasting conditions for both pH 1.2 (r = 0.9852, R^2^ = 0.9739) and pH 4.3 (r = 0.9896, R^2^ = 0.9756) media, compared with the fed conditions in pH 1.2 (r = 0.9706, R^2^ = 0.9415) and in pH 4.3 (r = 0.9804, R^2^ = 0.9598) (Fig. 6). All four IVIVC correlation coefficients are greater than their corresponding critical correlation coefficients which are r (degree of freedom 4, p = 0.001) = 0.9740 and r (degree of freedom 8, p = 0.001) = 0.8721.

**Fig. 7:**
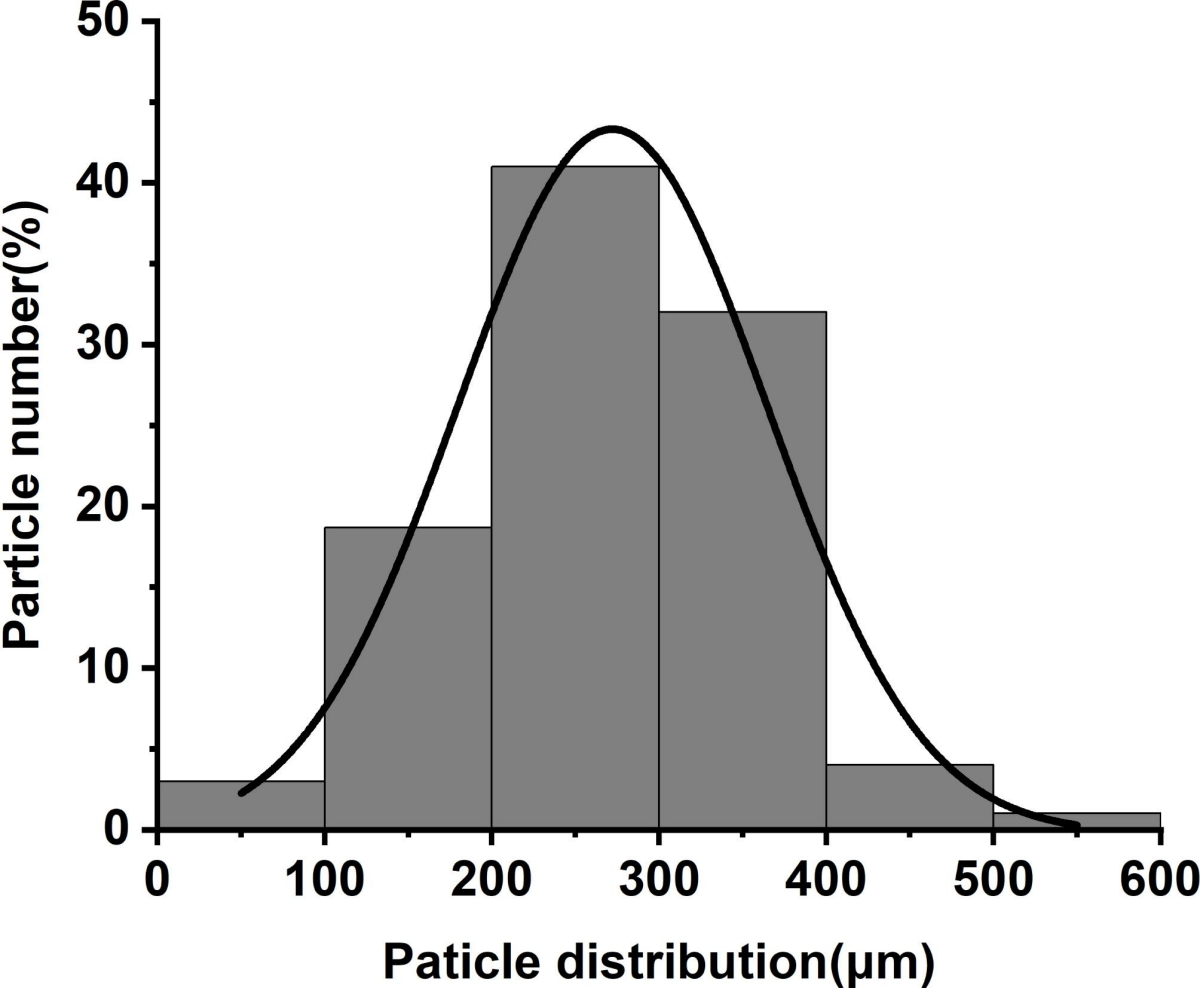
Correlation between in vitro released fraction and in vivo absorbed fraction of florfenicol from FSRGs in fasted and fed conditions. (A) In vitro released fraction in pH 1.2 and in vivo absorbed fraction of FSRGs in fasted condition. (B) In vitro released fraction in pH 4.3 and in vivo absorbed fraction of FSRGs in fasted condition. (C) In vitro released fraction in pH 1.2 and in vivo absorbed fraction of FSRGs in fed condition. (D) In vitro released fraction in pH 4.3 and in vivo absorbed fraction of FSRGs in fed condition

## Discussion

FFC is widely used in animal husbandry and aquaculture for its broad-spectrum bacteriostatic effect. In China, oral dosage forms of commercial FFC are all immediate-release. Most of them are a physical mixture of FFC and starch or monosaccharide, such as glucose. Ling and her colleagues prepared tilmicosin- and florfenicol-loaded hydrogenated castor oil-solid lipid nanoparticles using hot homogenization and ultrasonication method, which had a sustained-release effect both in vitro and in vivo [23]. Monostearate, which is highly hydrophobic, has been used in FSRGs formulation as sustained-release material, having similar properties as hydrogenated castor in Ling’s study [17, 23]. The release rate of florfenicol from granules can be controlled by the proportion of PEG 4000 whose mechanism is erosion-controlled drug release [21]. The findings of the formulation study showed that the release rate decreased with the increase of the proportion of monostearate. -In contrast, the release rate increased with the higher proportion of PEG 4000. Using starch as filler, had little influence on the release rate (data not shown). In this study, a FFC granules formulation that has the characteristics of sustained-release after oral administration was prepared by spray drying using readily available reagents monostearate, PEG 4000 and starch.

The spherical granules with high dispersibility and fluidity indicated that the spray drying parameters were appropriate for producing structured and spherical FSRGs (Fig. 3). High dispersibility and fluidity of the particles indicated that they could be mixed with large quantities of fodder in a uniform distribution.

The evaluation of in vitro release of formulation often takes place in the buffer of particular pH that stimulates the physiological conditions in vivo [14, 16, 18]. In this study, pH 1.2 and pH 4.3 were used to mimic the pH in the pig’s stomach and duodenum, respectively [24, 25]. The solubility of the florfenicol were 1938 µg/mL and 1860 µg/mL at pH 1.2 HCl solution and at pH 4.3 buffer (data not shown), respectively, indicating that the sink condition was fulfilled. Considering that the gastric emptying time of pigs is usually lower than 4 h, the last sampling point in pH 1.2 media (4 h) was different from that (8 h) of pH 4.3 buffer [25]. The cumulative drug release of FFC from the FSRGs was over 90.1% at pH 4.3 indicating an almost complete release in 8 h. Considering a minor difference in florfenicol solubility (about 4%) between pH 1.2 and pH 4.3 media, the difference in dissolution rate in different dissolution media may be due to the effect of pH on PEG 4000 [26]. The mechanism of FFC release from FSRGs was investigated by applying the release profiles at pH 1.2 and pH 4.3 dissolution media and fitted to 3 mathematical models: zero, first-order, and Higuchi models. Furthermore, the best fit was achieved using the Higuchi model (with the highest coefficient of determination) indicating that FFC released from FSRGs to the dissolution media through both diffusion and dissolution [27] (Table 1).

**Table 2:** Pharmacokinetic parameters of florfenicol after a single intravenous injection of florfenicol solution, oral administration of FSRGs in fasted and fed condition at a dosage of 20 mg/kg b.w. in swine. Data value were expressed as mean ± SD (n = 8)

PK parameters	FS-i.v. bolus	FSRGs-fasted condition		FSRGs-fed condition	
AUC_0-∞_(h??g/ml)	114.97 ± 12.90	128.32 ± 28.07		100.98 ± 15.03*	
K_el_(1/h)	0.21 ± 0.031	0.16 ± 0.066		0.209 ± 0.063	
T_1/2β_(h)	3.32 ± 0.50	5.23 ± 3.37		3.54 ± 0.88	
MRT(h)	4.52 ± 0.48	8.50 ± 1.16		7.35 ± 0.76*	
C_max_(µg/ml)	-	12.06 ± 1.94		12.49 ± 1.16	
Cl(L/h/kg)	0.18 ± 0.020	-		-	
V_d_(L/kg)	0.84 ± 0.095	-		-	
F(%)	-	~ 100%		87.83% ± 13.08	
		Median	Range	Median	Range
T_max_(h)	-	4.25	3.00–6.00	3.50	3.00–4.00

FS, florfenicol solution; i.v., intravenous; FSRGs, florfenicol sustained-release granules; AUC, the area under the concentration-time curve; K_el_, elimination rate constant; T_1/2β_, the elimination half-life; MRT, mean residence time; C_max_, maximal drug concentration; Cl, body clearance rate; V_d_, apparent volume of distribution; F, absolute bioavailability; T_max_, time to reach maximal drug concentration

*Statistical significances compared with FSRGs-fasted condition are p < 0.05;

When administering a drug orally, the intake of fodder is an important factor [28, 29]. To evaluate the effect of fodder intake on oral dosing, and to study the pharmacokinetics of FSRGs in pigs, we set three parallel groups rather than using other pharmacokinetic designs (time-cost consideration). In the first group florfenicol solution for administered intravenously, in the second and third, FSRGs were administered orally in fasting and non-fasting conditions, respectively. After a single i.v. dose of FS, the area under the concentration-time curve (AUC), elimination rate constant (K_el_), the elimination half-life (T_1/2β_), mean residence time (MRT), body clearance rate (Cl), and apparent volume of distribution (V_d_) were 114.97 ± 12.90 h·µg/ml, 0.21 ± 0.031 h − 1, 3.32 ± 0.50 h, 4.52 ± 0.48 h, 0.18 ± 0.020 L/h/kg, and 0.84 ± 0.095 L/kg, respectively. The T_1/2β_ and MRT values indicate that the drug was quickly eliminated from the plasma, which was in agreement with the findings reported by Liu, Jiang and Xu [15, 29, 30]. Significantly (p < 0.01) delayed T_max_ was observed in the curve of FSRGs in fasting and non-fasting conditions, which were 4.25 ± 1.16 h and 3.50 ± 0.53 h, respectively. Similarly, in Ling’s study, the T_max_ of florfenicol-loaded HCO-SLN (9 h) after oral administration was significantly prolonged compared with that of FFC API (1 h ) [17]. MRT values of FSRGs administered orally are nearly twice that of i.v., indicating the controlled-release characteristics of FSRGs in vivo. Interestingly, the T_max_ of FSRGs in fasting condition was longer than in fed condition, which may be due to the secretion of digestive juice that was stimulated by fodder and could react with pharmaceutical excipients in the FSRGs formulation. The C_max_ of FSRGs after oral administration in fasting and fed conditions were 12.06 ± 1.94 µg/ml and 12.49 ± 1.16 µg/ml, respectively (p > 0.05). The difference in T_1/2β_ values between FSRGs administrated orally (both in fasting and fed conditions) and i.v. were not significant (p > 0.05). Numerical difference of T_1/2β_ values in the fasting group, when compared with other groups, could be attributed to the experimental error such as variation among animal individuals. The AUC_0−∞_ were 128.32 ± 28.07 h·µg/mL, 100.98 ± 15.03 h·µg/mL, and the absolute bioavailability were ~ 100% and 87.83 ± 13.08% for FSRGs in fasting and fed conditions, respectively. AUC of FSRGs in fasting and fed conditions were significantly different (p < 0.05), fodder intake with FSRGs could decrease the bioavailability of the drug (Table 1).

FFC is a poorly soluble drug (1 mg/mL) which is also highly permeable (oral bioavailability > 85%). which is characteristic to Class II drugs, according to the Biopharmaceutical Classification System [16, 30, 31]. An in vitro - in vivo correlation (IVIVC) is expected if the dissolution rate in vivo is the rate-limiting step and similar to the in vitro rate [31]. A numerical deconvolution method (polynomial fitting, n = 2) was used to estimate the in vivo drug absorption, using the i.v. data as the unit impulse response function. The r values for the curves in both fasting and fed conditions were greater than the critical correlation coefficient (p < 0.001), indicating that a level A IVIVC was established. For the fed conditions, the curve was sigmoid at both pH 1.2 and 4.3, indicating that the in vivo absorption fraction was increased in the fasting condition over the in vitro drug release. This is the case when gastric and intestinal juice secretion increases during digestion. This could increase the rate of drug release from the FSRGs in vivo.

In summary, we produced a new florfenicol formulation that characterizes sustained-release in vitro and in vivo. Also, a level A IVIVC was established, indicating that dissolution profile of FSRGs was similar to that in the intestinal environment of the pig. We put forward that high-speed centrifugal spray drying had a great potential to produce commercial veterinary oral formulation on an industrial scale. Furthermore, to the best of our knowledge, our study produced the first oral FFC sustained-release granules, which could enable optimization of other oral veterinary sustained-release formulations. In addition, on the basis of this study, we plan to estimate the clinical effect of FSRGs in pigs using the pharmacokinetic-pharmacodynamic correlation method and try to develop more veterinary sustained-release preparations.

## Conclusions

FSRGs were successfully developed using a combination of monostearate, PEG 4000 and starch, in a spray drying process. Through diffusion and matrix erosion in pH 1.2 HCl solution and pH 4.3 acetate buffers, the granules could sustain drug release. Good in vitro and in vivo correlations were present in the fasting conditions, indicating that the in vivo profile of the FSRGs can be estimated on the basis of the in vitro drug release. In summary, the FSRGs we developed may be useful in developing other sustained-release formulations or in optimizing the oral dosage form of FFC. This study showed that the sustained-release formulation prepared using spray-drying may soon lead to the broader, commercial and industrialized use of FFC (in animal husbandry).

## Materials and methods

### Ethics statement

The experimental procedure was performed in accordance with the Regulations of Experimental Animal Administration rules of Laboratory Animal Center of South China Agricultural University and with the ARRIVE guidelines. The experiment was approved by the Ethics Committee of the Laboratory Animal Center of the South China Agricultural University.

### Materials

FFC reference standard (99.3% purity) was purchased from the China Institute of Veterinary Drug Control (Beijing, China). FFC active pharmaceutical ingredient (99.9% purity) was obtained from Haixiang Chuannan Pharmacy (Zhengjiang, China). High performance liquid chromatography (HPLC) grade acetic acid and acetonitrile were obtained from Macklin Biotechnology (Shanghai, China) and Fisher Scientific (Pittsburg, PA, USA), respectively. All other chemical reagents were analytical grade and purchased from commercial suppliers. Monostearate, polyethylene glycol 4000 (PEG 4000) and starch were purchased from pharmaceutical suppliers in Guangdong province, China.

### Preparation of florfenicol sustained-release granules

The formulation utilized a total mass of 2500 g and consisted of FFC, monostearate, PEG 4000 and starch, which were prepared in a weight ratio of 10:70:13:7. In brief, monostearate was heated to 85 ℃ with stirring followed by the gradual addition of other components and stirred at 100 rpm for 30 min at the same temperature. The mixture was then delivered to a high-speed centrifugal spray drying device (Zhengdian Biotechnology, Foshan, China). The main operational parameter values of the spray drying system were 500 kg/h (feed flow rate), 16,000 rpm (atomizer rotation speed), 16 ℃ (inlet temperature), and 28,000 m^3^/h (drying gas flow rate). The temperature of the feedstock solution from the feed tank to the atomizer was maintained at 85 ± 1 ℃ using the heater. Light microscopy and scanning electron microscopy were employed to structurally characterize the FSRGs collected in the product collection chamber.

The particle size distribution of FSRGs was measured using the mechanical sieving method with tailored sieves (sieve pore diameter from 100 to 600 μm). The instrument parameters of the mechanical sieve shaker (TJ-TAM, TECHIN, Tianjin, China) were 2000 times/min (vibration frequency) and 15 min (vibration time).

The stability of FSRGs was evaluated by the influencing factors test (high temperature of 40 ℃, 90% relative humidity, and 4500Lx ± 500Lx strong light exposure) for ten days and accelerated test (40 ± 2 ℃ and 75 ± 5% relative humidity) for six months using a drug stability test chamber (LHH-250GSP, Yiheng Instruments, Shanghai, China). The drug’s composition and characteristics of FSRGs were tested and analysed on the fifth and tenth day in the influencing factors test, and at the first, second, third, fourth and sixth month in the accelerated test, which were compared with the results obtained at the beginning of the tests.

### ***In vitro*** release tests

In vitro drug release was performed with the rotating basket method using an RCY-808 dissolution tester (Haiyida, Tianjin, China) according to Chinese Veterinary Pharmacopoeia using 900 mL of Hydrochloric acid solution at pH 1.2 and acetate buffer solution (0.1 M) at pH 4.3. The rotation speed was adjusted to 100 rpm and the dissolution temperature was 37 ℃. The reaction progress was monitored by sampling (5 mL) at 0.25, 0.5, 1, 2, 3 and 4 h in pH 1.2 media and 0.25, 0.5, 1, 2, 3, 4, 5, 6, 7 and 8 h in pH 4.3 media. Each test was recorded in triplicate (n = 3) and the analysis was performed considering mean ± standard deviation (SD).

After sampling, an equal volume of pre-warmed media at 37℃ was used to immediately replenish the reaction vessel. The measurement of FFC concentrations was performed using an HPLC system device (Shimadzu Co. Ltd., Japan) comprised a SIL-20 A autosampler set at 10 µL of injection volume, SPD-20 A ultraviolet/visible wavelength detector set at 224 nm, a CTO-10AS column oven set at 30 ℃ and two LC-20AT binary pumps. The column used was a Zorbax SB-C18 column (250 × 4.6 mm × 5 μm) (Agilent, Santa Clara, CA, USA). The isocratic mobile phase consisted of a mixture of acetonitrile-water-acetic acid at a ratio of 100:97:3 (v/v/v), and the flow rate was 1 mL/min.

### Kinetics of ***in vitro*** release

In vitro cumulative release was calculated according to the following equation:

1 $${Q}_{n}=\frac{{C}_{n}\cdot V+{\sum }_{i=n}^{n-1}{C}_{i}{V}_{i}}{m\omega }\cdot 100\% ({C}_{0} = 0,{V}_{0} = 0)$$

where Q_n_ is the cumulative drug release (CDR) at nh time; C_n_ and V stand for API concentration and volume of media at n time; C_i_ and V_i_ are used to define the concentration and volume of the sample at i time; Symbols m and ω denote mass and API content of FSRG in the in vitro release test (IVRT). The release mechanism of the drug was carried out by fitting CDR-to-time curve with the models (zero-order, first-order, Higuchi) using DDsolver software [32]. The correlation coefficient (r^2^) was employed to determine the best fit model for the drug release profile.

### Pharmacokinetic study of FSRGs in pigs

Twenty-four Landrace×Yorkshire pigs (male, 6-week-old ) weighing 15 ± 2 kg each were purchased from a farm in Yangjiang, Guangdong province, China. The animals were in optimal nutritional conditions and had free access to food and water when kept in an environmentally controlled breeding room for a week prior to the experiments at the Laboratory Animal Center of the South China Agricultural University. During the experiment, room humidity and temperature were controlled ranging from 55 to 70% and 23 ℃ to 27 ℃, respectively. The animals were divided into 3 groups of 8 and treated as follows: Group A received an *i.v*. bolus; Group B received FSRGs under fasting conditions; Group C received FSRGs under fed conditions. Oral administration under fasting conditions meant oral gavage with a stomach tube washed with physiological saline to flush the residual drug and fasted condition refers to the free access to the mixture of the drug and fodder.

Florfenicol solution for the *i.v*. bolus was filter-sterilized and prepared by dissolving 5 g florfenicol into 15 mL N-methyl pyrrolidone before adjusting the solution to 100 mL with 10% propylene glycol solution. A dose of 20 mg/kg body weight was administered to each group. The body weight data of pigs were obtained before each administration and the dose for each pig was adjusted accordingly. Pigs in each group were fasting for 8 h before and 2 h (Groups A and B) after administration and water was available *ad libitum*.

Blood samples (5 mL) from all animals were taken from jugular veins before drug administration and 5, 15, 30 and 45 min and 1, 2, 3, 4, 6, 8, 12, 16, 24, 36, 48, 60 and 72 h after *i.v.* bolus injection (Group A) and at 15, 30 and 45 min and 1, 2, 3, 4, 6, 8, 12, 16, 24, 36, 48, 60 and 72 h after oral administration (Groups B, and C ). To obtain plasma samples, blood was immediately collected into glass tubes containing heparin sodium and centrifuged at 4000 rpm for 10 min. The plasma sample was stored at -20 ℃ until analysis.

### Measurement of florfenicol concentration in plasma

An HPLC (same system mentioned above) method was designed on the basis of a previously reported method (16). Briefly, 1 mL of thawed plasma was placed into a 10 mL plastic cube followed by 0.5 mL of 0.1 M phosphate buffer at pH 7. FFC from plasma was extracted with ethyl acetate (3 mL) by two rounds of vortexing and centrifugation for 10 min. The organic layer was collected and evaporated under nitrogen in a 45 ℃ water bath. The residue was reconstituted in 0.5 mL acetonitrile solution (40%) and 20 µL was used for injection and the eluate was monitored at 224 nm. A reversed-phase column (Gemini C18, 250 × 4.6 mm,5 μm, Phenomenex, Torrence, CA, USA) was eluted with aqueous acetonitrile 72:28 at a flow rate of 1 mL/min at 30 ℃. The limit of determination (LOD) and limit of quantification (LOQ) were 0.02 µg/ml and 0.05 µg/ml, respectively. Florfenicol concentrations were determined using a calibration curve constructed in a range of 0.05 to 20 µg/mL (R^2^ = 0.9992). The inter- and intra-day variation for the determination in plasma ranged from 1.28 to 5.04% and 3.02–5.05%, respectively. The recovery of FFC in plasma ranged from 90.87 ± 3.3% to 101.04 ± 2.06%. The dilution effect was evaluated and samples that exceeded 20 µg/mL were diluted with acetonitrile solution (40%) obtained from blank plasma to adjust into the range of the calibration curve.

### Data analysis

Pharmacokinetic data were calculated and analyzed with Phoenix Winnonlin 8.1 (Certara USA, Inc.), using both compartmental and non-compartmental models. In vivo absorption was calculated using the deconvolution tool kit of Phoenix Winnonlin 8.1. In vitro and in vivo correlation analyses were performed using GraphPad Prism 8.0 (GraphPad Software, San Diego, California USA). All pharmacokinetic data were expressed as mean ± SD (n = 8). Statistical differences between groups were evaluated by applying the Student’s t-test and ANOVA using SPSS software Version 26.0 (IBM, Chicago, ILL, USA). P-values < 0.05 (P < 0.05) were considered to be statistically significant, and P-values < 0.01 (P < 0.01) were considered to be extremely significant. The schematic course of the present study is shown in Fig. 7.

## Data Availability

All data generated or analysed during this study are included in this published article.

## Abbreviations

FFC: Florfenicol
FSRGs: Florfenicol sustained-release granules
AUC: The area under the concentration-time curve
K_el_: Elimination rate constant
T_1/2β_: Elimination half-life
MRT: Mean residence time
Cl: Body clearance rate
V_d_: Apparent volume of distribution
SD: Standard deviation
CDR: Cumulative drug release
IVIVC: *In vitro - in vivo* correlation
